# Second Allogeneic Hematopoietic Stem Cell Transplantation for Hemophagocytic Syndrome with Engraftment Failure

**DOI:** 10.1007/s12288-022-01603-4

**Published:** 2022-12-06

**Authors:** Fan Jiang, Zhouyang Liu, Zikuan Guo, Juan Xiao, Nanhai Wu, Shifen Fan, Yan Yue, Jiao Chen, Yuan Sun

**Affiliations:** Department of Hematology and Oncology, Beijing Jingdu Children’s Hospital, 308 East Huilongguan Street, Changping District, Beijing, China

**Keywords:** Second allogeneic hematopoietic stem cell transplantation, Implantation failure, Hemophagocytic syndrome

## Abstract

This study aims to assess the efficacy of second allogeneic hemopoietic stem cell transplantation (allo-HSCT) for treating hemophagocytic syndrome with first engraftment failure. Among a total of 35 patients who underwent allo-HSCT between June 2015 and July 2021 for HLH, 10 patients who underwent a second HSCT following graft rejection were retrospectively analyzed. Various factors, such as the treatment course and outcome, the remission status, donor selection, and the conditioning regimen of patients before second allo-HSCT, were scrutinized for transplant-related complications and transplant-related mortality, as well as transplant outcomes. All the subjects have achieved complete donor engraftment, in which the neutrophils and platelets engraftment occurred in a median time of 12 d (range 10–19 d) and 24 d (range 11–97 d), respectively. Among the selected subjects, 20% of patients are diseased due to transplant-related thrombotic microangiopathy. Further, 90% of patients are diagnosed with aGVHD, in which 3 of them with grade I aGVHD, one patient with grade II aGVHD, two patients with grade III GVHD, and three patients with localized chronic GVHD. Moreover, 70% of patients showed signs of combined viral infections. Despite the complex symptoms, the overall survival rate is around 80%, with transplant-related mortality and the incidence of post-transplant GVHD of 20% and 60%, respectively. Together, our findings indicated that the second allo-HSCT showed great potential in treating hemophagocytic syndrome with engraftment failure.

## Introduction

Hemophagocytic syndrome (HPS), also referred to as hemophagocytic lymphohistiocytosis (HLH), is a group of clinical symptoms characterized by the uncontrolled activation and proliferation of the monocyte-macrophage system. This syndrome is characterized by hemophagocytosis and the secretion of various inflammatory cytokines, resulting in a group of disorders with similar clinical outcomes, such as abnormal coagulation mechanisms and abnormal organ function. In this vein, hematopoietic stem cell transplantation (HSCT) has been utilized as one of the prominent therapeutic strategies for addressing primary, relapsed, and refractory HLH. Among various transplanting procedures, reduced-intensity conditioning (RIC) transplantation has been recommended globally for reducing preconditioning toxicity and transplant-related mortality due to significantly improved overall efficiency over myeloablative conditioning (MAC) [1–4]. However, RIC-HSCT suffers from the main challenge of engraftment failure, requiring an additional HSCT procedure in patients with partial engraftment failure. In a case study, patients (n = 10) diagnosed with hemophagocytic syndrome and engraftment failure after the first HSCT at our center were subjected to a second HSCT.

## Experimental Section

### Subjects

Patients (n = 10, 6 male and 4 female participants) diagnosed with hemophagocytic syndrome and subsequent engraftment failure from June 2015 to July 2021 were considered for a second allo-HSCT in our hospital. The first HSCT in a patient (case 10) was performed in the other hospital, while the hematopoietic stem cell transplantation of remaining patients (cases 1–9) was completed in our hospital. Besides, the diagnostic criteria of all the patients for the hemophagocytic syndrome were fulfilled as revised in 2004 by the International Histiocyte Society [5]. The median age of the patient at the time of the first allo-HSCT was ca. 6.3 years (range 1.2–15.4 years), and the median time between two allo-HSCT cases was ca. 42.5 days (range 29–1364 days). The study was explained before obtaining the written informed consent according to the guidelines.

### Donors

All the donors were substituted while undergoing the second allo-HSCT to investigate the efficiency of the second graft. For example, the donor of the first allo-HSCT was not used for the second stem cell donor in any of the patients. All the patients had haploidentical stem cell donors in the first and second allo-HSCT. The stem cell donors in the first HSCT included a father in 8 cases, unrelated cord blood in 1 case, and an unrelated bone marrow stem cell bank in 1 case. During the second HSCT, the stem cells were collected from the corresponding mother donors in 7 cases and an unrelated bone marrow stem cell bank in 3 cases.

### First HSCT Setting

Prior to transplantation, all grafts were pretreated with RIC. The first HSCT setting in the 10 HLH subjects was designed as follows. Eight patients were transplanted with bone marrow and peripheral blood stem cells. Each subject was transplanted with the umbilical cord blood stem cells and peripheral blood stem cells, respectively. The pre-transplant disease status of 10 HLH subjects included a complete response in 3 patients, a partial response in 4 patients, and disease progression in 3 patients. To this end, 1 subject showed a refractory relapsed HLH, while the rest (n = 9) subjects displayed primary HLH (3 with unc13d mutation, 4 with XIAP mutation, 1 with stxbp2 mutation, and 1 with reduced cellular functions). The conditioning regimen in all the subjects was designed as follows: VP16 (600–900 mg/kg/min/4–6 d) + BU (0.8–1.2 mg/Kg/q6 h/3 d) + FLU(30–40 mg/m2/3 d) + ATG(8–8.5 mg/Kg/min/4 d) ± CTX (10 mg/kg/2 d) TBI (6–8 Gry/min/3d) + VP16 (600–900 mg/kg/min/4–6 d) + Ara-c (6 g/kg/min/4 d) + FLU (30–40 mg/m^2^/3 d) + ATG (7.5–8 mg/kg/min/4 d). In the situation of GVHD prophylaxis, 8 patients have received CSA + MMF + basiliximab regimen, and 2 patients with CSA + MMF + short-course regimen of methotrexate (MTX). To this end, the transplant-related complications, such as infections and early functional impairment, are displayed in Table 1. The median time to engraftment failure after transplantation was 21 d (range 14–31 d).

**Table 1:** Clinical data of patients with first allogeneic hematopoietic stem cell transplantation

Case	Gender	Age	Transplant (indication)	Disease state	Preprocessing regime	Donor type	Complication	Implantation failure time (d)
1	Male	1.5	Primary (XIAP)	AD	VP16 + BU + FLU + ATG + CTX	Father 6/10	No	+ 21
2	Male	1.7	Primary (XIAP)	PR	VP16 + BU + FLU + ATG	Father 5/10	No	+ 21
3	Female	7.5	Primary (UNC13D)	PR	TBI + VP16+ Ara-c + FLU + ATG	Father 7/10	CMV (blood)	+ 14
4	Female	12	Primary **(**STXBP2)	AD	VP16 + BU + FLU + ATG	Father 5/10	Liver damage	+ 21
5	Female	1.5	Primary **(**UNC13D)	CR	VP16 + BU + FLU + ATG	Umbilical Cord blood 9/10	Lung damage	+ 18
6	Male	3.6	Primary **(**XIAP)	CR	VP16 + BU + FLU + ATG	Bone marrow bank 8/10	No	+ 28
7	Female	6.8	Refractory & relapse	PR	VP16 + BU + FLU + ATG	Father 6/10	Epilepsy	+ 21
8	Male	15.4	Primary **(**UNC13D)	PR	TBI + VP16 + FLU + ATG + CTX	Father 5/10	Pulmonary intestinal infection	+ 21
9	Male	2.7	Primary **(**CD107a reduced)	AD	VP16 + BU + FLU + ATG + CTX	Father 5/10	No	+ 20
10	Male	1.2	Primary **(**XIAP)	CR	VP16 + BU + FLU + ATG	Father 5/10	Pulmonary infections	+ 31

### Second HSCT Setting

The second HSCT setting was designed as follows. Among the subjects, 3 patients were infused with mobilized peripheral blood stem cells, and 7 were infused with the combination of mobilized bone marrow and peripheral blood stem cells. As shown in Table 2, the conditioning regimen for the second HSCT was set as VP16 (300–600 mg/kg/min/4 d) + MEL (50–90 mg/m^2^/min/2 d) + FLU (30 mg/m^2^/3 d) + ATG (2.5–5 mg/Kg/min/4d) ± CTX (10 mg/kg/2 d). The calculated median time was 9.2 d (7.6–9.9 d). The mononuclear cells of 1 × 10^8^/kg were transfused during the second allo-HSCT, and the number of CD34^+^ cells was around 5.81 × 10^6^/kg (3.06–8.53 × 10^6^/kg). The engraftment was successful in all the patients, including cases 3 and 4, who developed grade III aGVHD due to their accumulation in the gut, liver, and skin. However, these patients (cases 3 and 4) died after 125 and 157 d of transplantation, respectively, due to a combined transplant-related TMA. The progress of the transplanted tissues of the remaining patients was followed up, regulating different complications that occurred after transplantation and administering related treatments.

**Table 2:** Clinical data of patients with secondary allogeneic hematopoietic stem cell transplantation

Case	T1 − T2 Time (d)	Disease status	Donor matching	Pre-processing regime	MNC X10^8^/kg	CD34+ X10^6^/kg	Granulocyte reconstruction day	Platelets reconstruction day	GVHD	Other complications	Follow-up time (month)	Survival condition
1	31	PR	Mother 6/10	VP16 + MEL + FLU +ATG	9.7	5.7	10	27	cGVHD	CMV (blood)	49	Positive
2	45	PR	Mother 6/10	VP16 + MEL + FLU +ATG	9.5	3.06	12	97	cGVHD	EBV (blood)	49	Positive
3	30	AD	Mother 5/10	VP16 + BU + Ara-C + FLU + ATG	8.8	6.22	11	16	III GVHD	TMA	4	Negative
4	29	AD	Mother 5/10	VP16 + MEL + FLU +ATG	7.6	5.8	11	73	III GVHD	TMA	5	Negative
5	30	CR	Bone marrow bank 9/10	VP16 + MEL + FLU +ATG	9	5.75	19	82	cGVHD	CMV (blood)	25	Positive
6	58	PR	Bone marrow bank 8/10	VP16 + MEL + FLU +ATG	8.9	8.53	12	18	I aGVHD	CMV (blood)	57	Positive
7	1364	CR	Mother 5/10	VP16 + MEL + FLU +ATG + CTX	9.4	6.9	10	11	II aGVHD	Cystitis	22	Positive
8	52	PR	Mother 5/10	VP16 + MEL + FLU +ATG	9.9	4.5	14	30	I aGVHD	CMV retinitis	16	Positive
9	40	CR	Mother 5/10	VP16 + MEL + FLU +ATG + CTX	9.67	5.82	12	15	I aGVHD	CMV (blood)	10	Positive
10	60	PR	Bone marrow bank 9/10	VP16 + MEL + FLU +ATG + CTX	8.57	6.42	15	21	No sign	CMV (blood)	3	Positive

### Monitoring of Engraftment

The preliminary blood tests were performed daily during the transplantation, including absolute neutrophil count (ANC), which indicated > 0.5 × 10^9^/L granulocytes for 3 consecutive days and ≥ 20 × 10^9^/L platelets for 7 consecutive days without platelet transfusion to achieve platelet engraftment indicators. Further, the donor chimerism was determined by the short-range tandem repeat–polymerase chain reaction (STR–PCR) of donor and recipient DNA. Finally, the disease status was determined by regular blood routine, bone marrow morphology, and cytogenetic (karyotype) examinations after transplantation.

### Complication Prevention

To prevent viral infection-related complications, a combination of ganciclovir and acyclovir was infused before and after transplantation. In addition, alprostadil was infused intravenously to prevent hepatic veno-occlusive disease and transplant-related thrombotic microangiopathy. Further, as a part of the preventive cure, the treatment options for protecting the liver, and stomach, inhibiting acid, avoiding emesis, as well as preserving the heart were prescribed in advance. More importantly, the patients were kept regularly hydrated and alkalinized. Moreover, the serum concentration of FK506 was detected at regular pre-determined intervals. In addition, immunoglobulins were infused after + 1 d, + 11 d, + 21d, and + 31 d of transplantation to prevent infection.

## Results

### Hematopoietic Reconstitution Landscape

Patients (n = 10) considered for a second allo-HSCT had hematopoietic reconstitution within a short period of time. The ANC value of around > 0.5 × 10^9^/L granulocytes and platelet count of ≥ 20 × 10^9^/L, within the median time of 12 d (10–19 d), and 24 d (11–97 d), respectively, were observed. All patients were subjected to the fully donor-chimeric STR testing results. Nonetheless, the different blood types were conveniently changed to donor blood types.

### GVHD Situations

A total of 6 subjects displayed the development of various grades of acute GVHD (aGVHD) conditions after the transplantation, including grade I in 3 cases, grade II in 1 case, and grade III in 2 cases, with a median onset time of 28 d (14–48 d) and an incidence rate of 60%. These aGVHD conditions occurred in different sites, such as the intestine (2 subjects), cutaneous (3 subjects), and hepatic (1 subject) regions. Nevertheless, these conditions were controlled in 4 patients after methylprednisolone administration. The aGVHD conditions were continued in two subjects, who were prescribed a second-line anti-GVHD therapeutic regimen for 3 months, resulting in no further progression and no GVHD-related death. The chronic localized GVHD3 occurred with a median time to the presentation of 145 d (range 120–190 d) and a cumulative incidence of 30%. These localized cGVHD conditions in the cutaneous region were significantly controlled after administering MTX.

### Other Transplant-Related Complications

After the second allo-HSCT, several virus-related infections were observed in 7 patients, including 5 patients with CMV viremia, 1 with EBV viremia, and 1 with CMV retinitis. However, these infections were significantly controlled by administering a fixed regimen of ganciclovir, foscarnet, and acyclovir-based anti-viral therapy. The hemorrhagic cystitis observed in 1 patient was substantially cured by alkalinization and hydration, further reducing the risk by treatment with mesna. To this end, the TMA condition observed in 2 patients was treated with plasmapheresis, defibrotide, and eculizumab, which, however, could not be controlled and led to death due to pulmonary hemorrhage.

### Efficacy and Outcome

The follow-up of this study in patients ended on July 25, 2021. The eventual outcome resulted in 8 cases of disease-free survival, and the rest 2 subjects died due to transplant-related TMA after 4 and 5 months of transplantation. Finally, the overall outcome of the design after the second HSCT included a median follow-up of 23.5 (3–57) months, with a disease-free survival rate of 100% (8/8 cases) and an overall survival rate of 80% (8/10 cases) during the follow-up.

## Discussion

Since its development in 2010, the HSCT approach has emerged as an innovative therapeutic option for treating HLH syndrome. Moreover, the improved awareness among clinicians may substantially allow them to treat more children using the HSCT approach. Indeed, the HSCT treatment at an earlier stage offers timely transplantation in remission, in terms of the continuous improvement of medical techniques, leading to better transplantation outcomes [6]. Based on their pathogenesis, the hemophagocytic syndrome is predominantly divided into two main groups, which are further divided into different subtypes, such as primary HLH and second HLH, as well as diverse genetic backgrounds and acquired pathogenic factors [7]. To this end, different treatment strategies for HLH have been developed based on the type and degree of etiology and pathogenesis. The first-line treatment currently recommended globally for HLH-94 or HLH-04 allows disease control by regulating immune cell activation, suppressing excessive immune responses, and clearing cytokine secretion. Considering these aspects, the allo-HSCT is the preferred treatment choice for primary HLH, as well as relapsed and refractory HLH [8]. The chief aim of the transplantation procedure is to remove the HLH-assisted immune disorders and restructure the typical immune system to achieve a thorough cure. However, the allo-HSCT approach may sometimes result in engraft failure, requiring a second HSCT. In this context, the second HSCT is performed to address the challenges associated with engraftment failure in primary HLH, refractory and relapsed HLH, and CNS HLH. Nevertheless, no significant reports demonstrated the efficacy of the second HSCT. In this study, we summarize our experience in terms of the prognosis of the second HSCT, prominently relating to the remission status, the choice of conditioning regimen, and the donor choice before transplantation.

In this study, the failure of the first implantation in HLH patients could be mainly mediated by immune factors. However, no HLA antibodies were detected in the patients. Moreover, the predominant rationale for choosing the mother as a haploidentical stem cell donor during the second transplantation was for three main reasons. First, there existed no other better donor, such as a sibling donor or a non-blood donor. Second, the patient’s condition was urgent, and no time for waiting to find a better donor. Three, the relevant investigations, including cytological tests, such as virus, CD107a, and XIAP, were performed for the present, resulting in normal values. Concerning the donor selection, most cases of first allo-HSCT were grafted from haploidentical donors compared with non-consanguineous donors due to the high convenience in receiving their donations than from the bone marrow bank donors. The pre-transplant disease progression often leads to a higher incidence of TRM [9, 10]. In addition, the recurrent pre-transplant HLH causing occult liver, lung, and other injuries, accounts for various VOD or non-infectious pneumonia during the transplantation [11–17]. Therefore, it is required to ensure a pre-transplantation remission state to minimize the notified complications. Previous reports indicated that pre-transplantation disease status in children with HLH was an independent prognostic factor for the later therapeutic outcomes. In this study, the remission status of patients was assessed during the preparation for second transplantation, classifying 70% of the cases as not in remission. Among them, one case of second transplantation with reduced-intensity conditioning effectively reduced the incidence of TRM.

In general, the second allo-HSCT is often considered unsafe as it is associated with adverse side effects and poor long-term survival rates in patients due to the high incidence of graft failure (GF), organ function toxicity, and infection-related complications. Schriber et al. [18] reported a clinical study of 122 unrelated patients with primary GF, who were treated with a second HSCT, demonstrating a 1-year long-term survival rate of 11% with a mortality rate of 75% after 100 days of transplantation. The incidence of GF after the second HSCT was reported as 31% (38/122), which could be referred to as the primary cause of death of the patient. In contrast, the increased infection in the patients could be another reason for the cause of death. This study pointed out the remission state of the underlying disease before the second transplantation as a significant contributor to the outcomes. Moreover, the authors demonstrated that acute and chronic GVHD risks were usually higher if the mother was applied as a donor. Nevertheless, our data showed that the probability of developing GVHD in the case of the mother as a donor was not significantly higher after a second HSCT.

## Conclusion

In summary, the study has explored the efficacy of second allo-HSCT for engraftment failure in HLH patients. The experimental results indicated a success rate of 80% of cases. Considering the need for longer study cycles, retrospective background analysis, and different patient types, further prospective studies are required to predict the relevant factors for the success of second allo-HSCT treatment. Together, we firmly believe that the second allo-HSCT showed great potential and as a feasible treatment strategy for engraftment failure after the first allo-HSCT for the hemophagocytic syndrome.

## Data Availability

The data is available upon reasonable request.

## References

[1] Marsh RA, Rao K, Satwani P (2013). Allogeneic hematopoietic cell transplantation for XIAP deficiency: an international survey reveals poor outcomes. Blood.

[2] Bhoopalan SV, Wlodarski M, Reiss U, Triplett B, Sharma A (2021). Reduced-intensity conditioning-based hematopoietic cell transplantation for dyskeratosis congenita: single-center experience and literature review. Pediatr Blood Cancer.

[3] Carl E, Rebecca M (2018). Reduced-intensity conditioning for hematopoietic cell transplant for HLH and primary immune deficiencies. Blood.

[4] Seo JJ (2015). Hematopoietic cell transplantation for hemophagocytic lymphohistiocytosis: recent advances and controversies. Blood Res.

[5] Henter J, Samuelsson-Horne A, Arico M (2002). Treatment of hemophagocytic lymphohistiocytosis with HLH-94 immunochemotherapy and bone marrow transplantation. Blood.

[6] Horne A, Wickström R, Jordan MB, Yeh EA, Naqvi A, Henter JI, Janka G (2017). How to treat involvement of the central nervous system in hemophagocytic lymphohistiocytosis. Curr Treat Options Neurol.

[7] Masood A, Wahab A, Iqbal Q, Davis J, Ehsan H, Hashmi H (2022). Efficacy and safety of allogeneic hematopoietic stem cell transplant in adults with hemophagocytic lymphohistiocytosis: a systematic review of literature. Bone Marrow Transpl.

[8] Eiichi Ishii (2016). Hemophagocytic lymphohistiocytosis in children: pathogenesis and treatment. Front Pediatr.

[9] Jong J (2015). Hematopoietic cell transplantation for hemophagocytic lymphohistiocytosis: recent advances and controversies. Blood Res.

[10] Marsh RA, Vaughn G, Kim MO, Li D, Jodele S, Joshi S (2010). Reduced-intensity conditioning significantly improves survival of patients with hemophagocytic lymphohistiocytosis undergoing allogeneic hematopoietic cell transplantation. Blood.

[11] Horne A, Janka G, Maarten Egeler R (2005). Haematopoietic stem cell transplantation in haemophagocytic lymphohistiocytosis. Br J Haematol.

[12] Meresse V, Hartmann O, Vassal G (1992). Risk factors for hepatic veno-occlusive disease after high-dose busulfan-containing regimens followed by autologous bone marrow transplantation: a study in 136 children. Bone marrow transpl.

[13] Baker KS, DeLaat CA, Steinbuch M (1997). Successful correction of hemophagocytic lymphohistiocytosis with related or unrelated bone marrow transplantation. Blood.

[14] Cesaro S, Locatelli F, Lanino E (2008). Hematopoietic stem cell transplantation for hemophagocytic lymphohistiocytosis: a retrospective analysis of data from the Italian association of pediatric hematology oncology (AIEOP). Haematologica.

[15] Durken M, Horstmann M, Bieling P (1999). Improved outcome in haemophagocytic lymphohistiocytosis after bone marrow transplantation from related and unrelated donors: a single-centre experience of 12 patients. Br J Haematol.

[16] Imashuku S, Hibi S, Todo S (1999). Allogeneic hematopoietic stem cell transplantation for patients with hemophagocytic syndrome (HPS) in Japan. Bone Marrow Transpl.

[17] Hamidieh AA, Zahra Pourpak (2014). Fludarabine-based reduced-intensity conditioning regimen for hematopoietic stem cell transplantation in primary hemophagocytic lymphohistiocytosis. Eur J Haematol.

[18] Schriber J, Agovi MA, Pasquini MC (2010). Second unrelated donor hematopoietic cell transplantation for primary graft failure. Biol Blood Marrow Transpl.

